# Fetal loss and maternal serum levels of 2,2',4,4',5,5'-hexachlorbiphenyl (CB-153) and 1,1-dichloro-2,2-bis(*p*-chlorophenyl)ethylene (p,p'-DDE) exposure: a cohort study in Greenland and two European populations

**DOI:** 10.1186/1476-069X-9-22

**Published:** 2010-05-10

**Authors:** Gunnar Toft, Ane M Thulstrup, Bo A Jönsson, Henning S Pedersen, Jan K Ludwicki, Valentyna Zvezday, Jens P Bonde

**Affiliations:** 1 Department of Occupational Medicine, Aarhus University Hospital, Aarhus, Denmark; 2 Division of Occupational and Environmental Medicine and Psychiatric Epidemiology, Lund University Hospital, Lund, Sweden; 3 Centre for Arctic Environmental Medicine, Nuuk, Greenland; 4 Department of Environmental Toxicology, National Institute of Hygiene, Warsaw, Poland; 5 Kharkiv National Medical University, Kharkiv, Ukraine; 6 Department of Occupational Medicine, Copenhagen University Hospital Bispebjerg, Copenhagen, Denmark

## Abstract

**Background:**

In the present study, the aim is to examine the risk of fetal loss related to environmental 2,2',4,4',5,5'-hexachlorobiphenyl (CB-153) or 1,1-dichloro-2,2-bis(*p*-chlorophenyl)ethylene (p,p'-DDE) exposure.

**Methods:**

We related LC/MS/MS measurements of CB-153 and p,p'-DDE in serum samples to interview-data on previous fetal loss in populations of pregnant women from Poland, Ukraine and Greenland.

**Results:**

In total, 1710 women were interviewed, and 678 of these had at least one previous pregnancy. The risk of ever experiencing a fetal loss increased at higher levels of CB-153 and p,p'-DDE exposure, with an adjusted odds ratio (OR) of 2.4; confidence interval (CI) (1.1-5.5) for CB-153>200 ng/g lipid compared to 0-25 ng CB-153/g lipid and OR of 2.5 CI (0.9-6.6) for p,p'-DDE>1500 ng/g lipid compared to 0-250 ng DDE/g lipid. However, no clear dose response associations were observed. The results further suggest that high level of organochlorine serum concentrations may be related to repeated loss.

**Conclusions:**

The risk of fetal loss may increase at higher levels of CB-153 and p,p'-DDE exposure, although lack of dose response and inconsistencies between countries did not allow for firm conclusions.

## Background

Exposure to persistent organochlorine pollutants (POP)s are ubiquitous among humans. Since the production and use of POPs are banned in most countries, the main exposure route for the general population is through consumption of food contaminated with POPs from earlier release into the environment. The compounds bioaccumulate in the food chain and are occurring in relative high concentrations in fatty fish and sea mammals. Some native populations, including Inuits rely on seafood as a major dietary source and are, therefore, exposed to high levels of POPs [1]. However, in some tropical areas, organochlorine pesticides as for example dichlorodiphenyltrichloroethane (DDT) are still used, and in these geographical areas, pesticide applicators as well as people living in pesticide sprayed houses are exposed to several fold higher levels of DDT than the general population in the rest of the world [2].

High level of POP exposure has previously been indicated to be associated with adverse reproductive outcomes in some human studies. After the accidental exposure to dioxins and polychlorinated biphenyls (PCBs) through cooking oil contamination in Taiwan and Japan, spontaneous abortions and stillbirths were about 2-fold more frequently observed - especially in the first 10 years after the accident [3,4]. Using data from the Collaborative Perinatal Project in the USA with blood samples collected in 1959-1965, Longnecker and colleagues found that the high level of dichlorodiphenyldichloroethylene (DDE) the general population was exposed to in the 60'es was associated to an increased risk of fetal loss, with an odds ratio of 1.4 per 60 μg/l increase in DDE [5]. In India, a hospital-based case-referent study reported a high organochlorine pesticide concentration (sum of benzene hexachloride (BHC), Lindane, Aldrin, p,p'-DDE, p,p'-DDD and p,p'-DDT) in the blood of women having spontaneous abortions compared to women with full term babies [6]. Also, other retrospective studies of the organochlorine level in patients after spontaneous abortions indicate that organochlorines may play a role in spontaneous abortions [7-9]. On the other hand, several studies from Australia, Japan, Sweden and the United States were not able to demonstrate adverse effects of organochlorines on fetal loss [10-14]. Recently, a large and well designed prospective study was conducted to address the effects of DDT on spontaneous abortions by recruiting female Chinese textile workers before they became pregnant, and by following the pregnancy outcome [15]. In this study, the odds for spontaneous abortion increased with increasing exposure to total DDT and p,p'-DDE.

Thus, a number of predominantly smaller studies have been performed among women with high exposure to organochlorine contaminants, but risk of fetal loss related to present day exposure levels experienced by European and Arctic populations has not been described. Therefore, with the material collected from a European study of fertility in relation to organochlorine exposure http://www.inuendo.dk, we aim at evaluating whether exposure to organochlorines increases the risk of fetal loss in low- to medium-level exposed populations.

## Methods

### Subjects and sampling

Between June 2002 and May 2004, we recruited pregnant women and their male spouses in Greenland, Kharkiv (Ukraine) and Warsaw (Poland). All women, who previously had been pregnant, were asked about the number of previous miscarriages, the number of stillbirths and the total number of previous pregnancies and had a blood sample drawn. At the time of interview and blood sampling, the women were on average 24 weeks pregnant in Greenland and Kharkiv, and 33 weeks pregnant in Warsaw. A detailed description of recruitment process and participation has been given elsewhere [16] Briefly, the same questionnaire was translated to the native languages and used for participants from Greenland, Kharkiv and Warsaw. Pregnant couples were consecutively enrolled during antenatal care visits in Greenland and Kharkiv and at maternity schools in Warsaw. The participants had to be born in the country of the study and at least 18 years to be eligible for the study. Altogether, 1710 couples were interviewed. Depending on different procedures for recruitment, the participation rate differed among the four countries with low rates in Kharkiv (26%), but higher in Warsaw (68%) and among the Inuits from Greenland (90%).

The local ethical committees representing all participating populations approved the study, and all subjects signed an informed consent.

### Outcome

Of the 1710 interviewed women, 678 had experienced at least one previous pregnancy (429 Inuits, 49 from Warsaw and 200 from Kharkiv). All previeously pregnant women were asked about the number of previous miscarriages, the number of stillbirths and the total number of previous pregnancies. Fetal loss was defined at the individual level as the number of previous self-reported miscarriages or stillbirths, and ever experiencing a fetal loss was defined as 1 or more self-reported miscarriages or stillbirths.

### Collection of blood samples

Blood samples from the pregnant women were drawn from a cubital vein into 10 ml vacuum tubes for serum collection without additives (Becton Dickinson, Moylan, France). After cooling to room temperature, the tubes were centrifuged at 4000 g for 15 min. Serum was transferred with ethanol rinsed Pasteur pipettes to ethanol rinsed brown glass bottles (Termometerfabriken, Gothenburg, Sweden). A piece of aluminium foil was placed on top of the bottles, which were then sealed. Sera were stored at -20°C until shipment, but it was accepted to keep it in refrigerator for up to four days before freezing.

### Determination of CB-153 and p,p'-DDE in serum

All analyses of CB-153 and p,p'-DDE in serum were performed at the Department of Occupational and Environmental Medicine in Lund, Sweden, applying solid phase extraction using on-column degradation of the lipids and analysis by gas chromatography-mass spectrometry. The analyses of CB-153 and p,p'-DDE were part of the Round Robin inter-comparison quality control program (Professor Hans Drexler, Institute and Out-Patient Clinic for Occupational, Social and Environmental Medicine, University of Erlangen-Nurenberg, Germany) with analysis of results found to be within the tolerance limits. The tolerance limits were set as plus/minus three times the standard deviation of the results from a number of reference laboratories. Levels of detection and coefficients of variation have been described in detail elsewhere [1]. The distribution of exposures on this population have been described in detail previously [1].

### Determination of lipids by enzymatic methods

Serum concentrations of triglycerides and cholesterol were determined by enzymatic methods. The total lipid concentration in serum (g/L) was estimated by the following method: total = 0.96+1.28 (triglycerides+cholesterols) [17].

### Potential confounders

In the interview, the women were asked about several potential confounders, including age at conception of the present pregnancy, parity, prior number of induced abortions, smoking (yes/no) and pre-pregnancy height and weight. Women were considered to have had urogenital inflammation, if they stated to have ever been diagnosed with one of the following diseases: pelvic inflammatory disease, gonorrhoea or chlamydia infection, other sexually transmitted diseases, or pelvic infections after a previous pregnancy. Furthermore, urogenital diseases was defined as operations due to ovarian cysts, fibroids, myomas, endometriosis, or other operations of the fallopian tubes or ovaries, or chemical or radiation therapy because of urogenital cancer. Women with either thyroid disease or diabetes mellitus were considered as having a chronic disease.

We considered age at conception of the current pregnancy (continuous), BMI (continuous), smoking (yes/no), alcohol consumption (>14 alcoholic drinks/weeks; yes/no), number of previous pregnancies (continuous), previous induced abortions (none or one/two or more), number of live-born children (continuous), urogenital surgery (yes/no), urogenital inflammation (yes/no), and chronic diseases (yes/no) as potential confounders, and included these variables in the adjusted analyses, due to previously reported strong associations of these covariates to spontaneous abortion and/or organochlorine exposure.

### Statistics

The probability of ever experiencing fetal loss in five chosen categories of CB-153 and p,p'-DDE exposure was evaluated by means of logistic regression analysis, with the lowest exposure category as the reference level. In addition, logistic regression analysis of risk of fetal loss in relation to organochlorine exposure level (log transformed) was performed, and data from these analyses are given as the odds ratio per unit increase on the log scale of the exposure (per 1 log unit). Data are presented both as only adjusted for number of previous pregnancies in the models containing more than one previous pregnancy and fully adjusted for the above mentioned potential confounders. We include a model with only one previous pregnancy, since the number of previous pregnancies may affect the fetal loss rate, and although we adjust for this, the adjustment cannot exclude residual confounding. Furthermore, since exposure is measured in the present pregnancy, using women with one previous pregnancy would give less misclassification of exposure.

To evaluate whether not only the chance of ever experiencing a loss, but also increasing number of fetal losses was associated to POP exposure, we performed a general linear regression analysis on the association between POP exposure level and number of losses classified as 0, 1 and 2 or more fetal losses. The models were adjusted for potential confounders mentioned above. For individual comparisons of exposure level in the three specified groups, we used the group with no fetal losses as reference and compared that group to the 1-2 and 2 or more losses, respectively, by least squares means tests.

Homogeneity of associations was checked by inclusion of a fetal loss by country interaction terms in the models. We found no statistical significant indications of interaction, and therefore combined models without the interaction term are presented. For both the logistic regression model and the general linear regression model, the final model combined data from the three populations adjusted for population.

All statistical analysis was performed using SAS ver. 9.1 (SAS Institute Inc., Cary, NC, USA).

## Results

The three populations included in the present study differed as regards to miscarriage rate in earlier pregnancies, and especially the Polish women from Warsaw had a high rate of miscarriages and stillbirths (Table 1). The exposure level varied greatly between populations; the lowest CB-153 exposure was seen in Warsaw, and the exposure was also relative low in Kharkiv, whereas about 10-fold higher mean exposure level was found in Greenland. The p,p'-DDE exposure level was almost even in Warsaw and Greenland and about 2-fold higher in Kharkiv [1]. The potential confounders mentioned in Table 1 also differ somewhat between populations, and especially urogenital inflammation, smoking, drinking more than 14 drinks/week and induced abortion rate was higher in Greenland compared to the other populations. Furthermore, the population from Warsaw seemed to have a higher level of urogenital diseases and chronic diseases.

**Table 1:** Characteristics of the included populations

	Greenland n = 429	Kharkiv n = 200	Warsaw n = 49
**Reproductive outcomes**			

Miscarriages a	23.6%	18.0%	61.4%

Stillbirths a	1.0%	0.4%	4.0%

Induced abortions b	32.7%	7.8%	0%

Ectopic pregnancies b	1.0%	0.5%	1.0%

Fetal loss c	24.7%	18.1%	65.5%

**Exposure**			

CB-153 ng/g lipid mean (SD)	182 (229)	34 (19)	11 (9)

DDE ng/g lipid mean (SD)	451 (463)	783 (494)	433 (304)

**Potential confounders**			

Age mean (SD) d	28 (6)	29 (5)	30 (4)

BMI (kg/m^2^) mean (SD)	24.4 (4.4)	22.4 (3.1)	22.2 (3.6)

Previous pregnancies mean (SD)	2.7 (2.0)	1.8 (1.2)	1.3 (0.5)

Urogenital inflammation	86.7%	8.5%	12.2%

Urogenital disease	8.6%	8.5%	26.5%

Smoking e	86%	36%	37%

> 14 drinks of alcohol/week	11%	0.5%	0%

Chronic disease	2.6%	8.5%	12.2%

More than two previous induced abortions	42%	5.5%	0%

a. The average proportion of miscarriages and stillbirths outcomes out of total number of previous pregnancies per woman (except induced abortions and ectopic pregnancies).

b Average percentage of total pregnancies per woman ending as induced abortion or ectopic pregnancy.

c Miscarriages and stillbirths.

d Age at last planned pregnancy.

e Before last pregnancy.

In the combined analysis across countries, the risk of ever having experienced a fetal loss is higher at all CB-153 exposure categories compared to the reference level (0-25 ng/g lipid) although only significantly significant different at the highest exposure level (>200 ng/g lipid CB-153) with an adjusted odds ratio in the analysis across populations of 2.4 CI 1.1-5.5 (Table 2). The analysis on CB-153 as a continuous variable indicates a dose dependent increase of 1.2 CI 1.0-1.5 per log unit increase of CB-153. In the three separate countries, adjusted fetal loss was not statistically significantly associated to increasing CB-153 exposure, although most of the odds ratios for fetal loss exceeded unity. When restricting the analysis to the persons who only have had one previous pregnancy, the overall estimated odds ratios were approximately the same, but the confidence intervals were wider (Table 2). However, among the population from Poland, the pregnancy adjusted odds for fetal loss and odds for fetal loss among women with only one previous pregnancy increased with increasing CB-153 exposures.

**Table 2:** Distribution between exposure groups and risk of ever experiencing a pregnancy loss in categories of CB-153 exposure in relation to the lowest CB-153 exposure group

	CB-153 (ng/g lipid)					
	**0-25**	**25-50**	**50-100**	**100-200**	**>200**	**Continuous** **(Per 1 log unit)**

**Greenland**						

Number of women with at least one previous pregnancy	33	52	113	115	116	429

Number of women ever who ever experienced a fetal loss	12	24	48	49	68	201

Number of women with only one previous pregnancy	13	28	41	37	30	149

Number of women experiencing fetal loss in one pregnancy	2	8	9	5	9	33

Preg adj. odds ratio. (95%CI) a	1	1.7 (0.7-4.5)	1.1 (0.5-2.6)	1.0 (0.4-2.4)	1.6 (0.7-3.7)	1.1 (0.9-1.3)

Adj. odds ratio (95%CI) b	1	1.6 (0.4-6.2)	1.3 (0.4-4.3)	0.8 (0.2-2.8)	1.6 (0.5-5.5)	1.0 (0.7-1.3)

Adj. OR one prev. pregnancy	1	1.2 (0.2-8.5)	0.8 (0.1-4.9)	0.8 (0.1-5.6)	1.7 (0.3-11.7)	1.2 (0.7-2.0)

**Kharkiv**						

Number of women with at least one previous pregnancy	81	85	33	1	0	200

Number of women ever who ever experienced a fetal loss	16	23	10	1	0	50

Number of women with only one previous pregnancy	46	50	21	1	0	118

Number of women experiencing fetal loss in one pregnancy	7	9	4	1	0	21

Preg adj. odds ratio. (95%CI) a	1	1.5 (0.7-3.2)	1.8 (0.6-4.7)	-	-	1.6 (0.9-2.8)

Adj odds ratio (95%CI) b	1	1.6 (0.7-3.7)	1.7 (0.6-5.2)	-	-	1.4 (0.7-2.8)

Adj. OR one prev. pregnancy	1	1.2 (0.4-4.1)	1.3 (0.3-6.2)	-	-	1.0 (0.4-2.4)

**Warsaw **n						

Number of women with at least one previous pregnancy	45	4	0	0	0	49

Number of women ever who ever experienced a fetal loss	30	4	0	0	0	34

Number of women with only one previous pregnancy	39	2	0	0	0	41

Number of women experiencing fetal loss in one pregnancy	25	2	0	0	0	27

Preg adj. odds ratio. (95%CI) a	1	-	-	-	-	2.5 (1.0-5.9)

Adj odds ratio (95%CI) b	1	-	-	-	-	1.4 (0.1-78.8)

Adj. OR one prev. pregnancy	1	-	-	-	-	4.7 (1.2-18.3)

**Combined **c						

Number of women with at least one previous pregnancy	159	141	146	116	116	678

Number of women ever who ever experienced a fetal loss	58	51	58	50	68	285

Number of women with only one previous pregnancy	98	80	62	38	30	308

Number of women experiencing fetal loss in one pregnancy	34	19	13	6	9	81

Preg adj. odds ratio (95%CI) a	1	1.7 (1.0-3.0)	1.4 (0.8-2.6)	1.3 (0.7-2.5)	1.9 (1.0-3.8)	1.2 (1.0-1.4)

Adj odds ratio (95%CI) b	1	1.9 (0.9-3.9)	1.9 (0.9-4.0)	1.4 (0.6-3.2)	2.4 (1.1-5.5)	1.2 (1.0-1.5)

Adj. OR one prev. pregnancy	1	1.6 (0.6-4.2)	1.6 (0.5-4.7)	1.3 (0.3-5.1)	2.5 (0.7-9.2)	1.4 (0.9-2.0)

a Adjusted for number of previous pregnancies. b Adjusted for age, number of previous pregnancies, smoking, alcohol consumption, urogenital diseases, urogenital infections, chronic diseases, body mass index and two or more previous induced abortions. c Calculated odds ratios are all adjusted for country.

"-" refers to missing results due too low number of individuals in the strata to perform the desired analyses

A similar pattern of increased risk of fetal loss at the higher levels of p,p'-DDE exposure was observed (Table 3), although the adjusted odds ratios for the analysis across populations was not statistically significant at higher levels of exposure compared to the lowest level (0-250 ng/g lipid). Also here, the highest odds ratio was found at the highest exposure level (2.5 CI 0.9-6.6). The analysis on p,p'-DDE as a continuous variable did not indicate a consistent dose dependent association across populations (OR 1.0 CI 0.8-1.3). The adjusted odds ratios restricted to persons with one previous pregnancy gave similar estimates, but with wider confidence intervals. Analyses stratified on countries indicated that the risk of spontaneous abortions was increased at the highest level of DDE present in Greenland. However, this result was only statistically significant among data restricted to one previous pregnancy.

**Table 3:** Distribution between exposure groups and risk of ever experiencing a pregnancy loss in categories of p,p'-DDE exposure in relation to the lowest DDE exposure group.

	p,p'-DDE (ng/g lipid)					
	**0-250**	**250-500**	**500-1000**	**1000-1500**	**>1500**	**Continuous** **(Per 1 log unit)**

**Greenland**						

Number of women with at least one previous pregnancy	179	121	90	22	17	429

Number of women ever who ever experienced a fetal loss	72	56	51	10	12	201

Number of women with only one previous pregnancy	73	42	26	7	1	149

Number of women experiencing fetal loss in one pregnancy	14	10	9	0	0	33

Preg adj. odds ratio (95%CI) a	1	1.2 (0.7-1.9)	1.7 (1.0-2.9)	0.8 (0.3-2.2)	1.6 (0.5-5.4)	1.1 (0.9-1.3)

Adj odds ratio (95%CI) b	1	0.9 (0.4-1.9)	1.3 (0.5-2.9)	0.8 (0.2-3.8)	3.2 (0.6-15.8)	1.0 (0.7-1.3)

Adj. OR one prev. pregnancy	1	1.8 (0.6-5.2)	4.2 (1.2-14.1)	-	-	1.1 (0.7-1.8)

**Kharkiv**						

Number of women with at least one previous pregnancy	4	54	95	33	14	200

Number of women ever who ever experienced a fetal loss	2	12	23	8	5	50

Number of women with only one previous pregnancy	2	30	55	21	10	118

Number of women experiencing fetal loss in one pregnancy	1	5	10	2	3	21

Preg adj. odds ratio (95%CI) a	-	1 c	1.4 (0.9-2.1)	0.9 (0.5-1.9)	1.8 (0.7-4.2)	1.0 (0.5-1.8)

Adj odds ratio (95%CI) b	-	1 c	1.2 (0.5-2.8)	0.8 (0.2-2.6)	1.4 (0.3-6.0)	0.8 (0.4-1.5)

Adj. OR one prev. pregnancy	-	1 c	0.8 (0.2-2.5)	0.4 (0.1-2.2)	1.6 (0.3-9.1)	0.7 (0.3-1.7)

**Warsaw**						

Number of women with at least one previous pregnancy	13	23	11	1	1	49

Number of women ever who ever experienced a fetal loss	7	18	8	1	0	34

Number of women with only one previous pregnancy	12	19	9	0	1	41

Number of women experiencing fetal loss in one pregnancy	7	14	6	0	0	27

Preg adj. odds ratio (95%CI) a	1	3.0 (0.7-13.1)	2.2 (0.4-12.4)	-	-	1.8 (0.7-4.5)

Adj odds ratio (95%CI) b	1	-	-	-	-	1.0 (0.8-1.3)

Adj. OR one prev. pregnancy	1	3.5 (0.4-28.8)	3.5 (0.3-43.3)	-	-	2.8 (0.7-10.7)

**Combined **d						

Number of women with at least one previous pregnancy	196	198	196	56	32	678

Number of women ever who ever experienced a fetal loss	81	86	82	19	17	285

Number of women with only one previous pregnancy	87	91	90	28	12	308

Number of women experiencing fetal loss in one pregnancy	22	29	25	2	3	81

Preg adj. odds ratio (95%CI) a	1	1.2 (0.8-1.9)	1.5 (0.9-2.4)	1.0 (0.5;2.2)	1.9 (0.8-4.8)	1.0 (0.8-1.2)

Adj odds ratio (95%CI) b	1	1.0 (0.6-1.7)	1.3 (0.7-2.3)	0.9 (0.4-2.2)	2.5 (0.9-6.6)	1.0 (0.8-1.3)

Adj. OR one prev. pregnancy	1	1.4 (0.7-2.9)	1.6 (0.7-3.7)	0.5 (0.1-2.3)	1.7 (0.3-8.1)	1.1 (0.8-1.6)

a Adjusted for number of previous pregnancies. b Adjusted for age, number of previous pregnancies, smoking, alcohol consumption, urogenital diseases, urogenital infections, chronic diseases, body mass index and two or more previous induced abortions. c Due to the low number of individuals in the lowest strata in Kharkiv, the two lowest strata is combined as a reference in the analysis stratified on country. d Calculated odds ratios are all adjusted for country.

"-" refers to missing results due too low number of individuals in the strata to perform the desired analyses

When evaluating the number of fetal losses in relation to CB-153 and p,p'-DDE, the number of fetal losses increased with increasing CB-153 and p,p'-DDE level (Table 4). However, when looking at the individual countries, no association of PCB or DDE exposure and number of fetal loss could be detected in the Polish population.

**Table 4:** Geometric mean CB-153 or p,p'-DDE adjusted for age and pregnancies and 95% confidence grouped by pregnancy losses

	No fetal loss	1-2 fetal losses	>2 fetal losses
CB-153 (ng/g lipid)			

Greenland	102 (88;118)	113 (97;132)	208 (126;343)^a^

Ukraine	28 (26;31)	31 (26;36)	41 (23;73)

Poland	9 (5;18)	9 (7;13)	5 (1;36)

Combined	30 (26;35)	35 (31;41)	61 (41;89)^a^

p,p'-DDE (ng/g lipid)			

Greenland	265 (227;308)	291 (247;342)	510 (304;855)^a^

Ukraine	688 (630;753)	628 (532;741)	892 (514;1550)

Poland	537 (304;948)	316 (242;412)	61 (13;299)

Combined	414 (358;478)	451 (389;523)	713 (482;1055)^a^

The estimated means is controlled for age, number of previous pregnancies, number of live born children, smoking, alcohol consumption, urogenital diseases, urogenital infections, chronic diseases, body mass index and previous induced abortions

^a ^Indicates significant higher level of POP level compared to the no fetal loss group (Least squares means test).

## Discussion

The present paper provide limited evidence that the risk of experiencing fetal loss and the number of fetal losses are increased at higher level of exposure to the organochlorine compounds CB-153 and p,p'-DDE. The data did not show significant dose-response associations in the combined estimates across countries, although the odds ratios suggested a weak association, and a higher risk of fetal loss was found at high level of CB-153 in the combined estimate.

Only few significant associations were found in the analysis based on the single countries.

An increased risk of fetal loss in relation to organochlorine exposures has previously been observed in cohorts exposed to relative high or medium level of organochlorines [3-5,15], whereas other studies including populations at medium or low exposure levels were not able to detect an effect of organochlorines on fetal loss [10-14]. It should be noted that the latter studies included small study populations or exposure assessment based on fish consumption.

Our study included populations at low or medium level of exposure and represented the range of present exposure found in the general populations in European and Inuit populations. Although not statistically significant at all separate study populations, our results adds to the studies, suggesting that organochlorine exposure increases the risk of fetal loss, and our results suggest that even at relative low level of exposure organochlorines may affect the risk of fetal loss.

Results from animal studies support that organochlorines may cause spontaneous abortions and still births with the most sensitive species being the rhesus monkey [18], whereas rats and other mammals seems to be less sensitive to organochlorine exposure [19].

An increase in risk of fetal loss was not only observed in the populations with high exposure, but also in the Warsaw representing low CB-153 exposure. This may indicate that other compounds correlated to CB-153 and p,p-'DDE exposure may be involved in the observed effects. CB-153 correlated very well (r = 0.9) with previous reported total PCB concentration in plasma and serum from Swedish subjects [20,21], and with the dioxin and furan equivalent (TEQ) in plasma [22](r = 0.5). In the present study, CB-153 and p,p'-DDE are highly correlated [1], and therefore we are not able to identify if these compounds have independent effects on fetal loss.

A major limitation of the present study is the potential misclassification of both outcome and exposure. Previous studies have shown that when women are asked about previous spontaneous abortions, about 75% of the cases are recalled [23], and similar results are found in validation studies, comparing recalls and medical records in of fetal loss [24]. In our case, this would most likely cause non-differential misclassification, since the exposure level is not known, resulting in attenuation of any true effects. Therefore, the risk of fetal loss in relation to organochlorine exposure may be even stronger than reported in the present paper, whereas the potential misclassification is unlikely to have caused false positive associations.

Also, some misclassification of exposure is expected, since the exposure is measured at the present pregnancy, but the relevant time period of exposure is during previous pregnancies. Since the half-lives of CB-153 and p,p'-DDE is up to 10 years, the present exposure may be a reasonably good estimate of exposure during the recent years, when previous children were born. However, one special problem associated to exposure misclassification is that a previous pregnancy ending as a fetal loss does not reduce the organochlorine level as much as a pregnancy ending as a live birth, where the organochlorine level is reduced during lactation. This may induce a false association between organochlorine exposure and previous spontaneous abortions, as discussed in detail in [5]. The main analysis in our study differ from the study by Longnecker et al [5] by using the women as the statistical unit and not the individual pregnancies. Therefore, we were able to include number of live born children as a proxy for lactational loss of POPs and thereby adjust for a marker of reduction of organchlorines due to lactation. Unfortunately, direct lactation information was not available. Including number of live born children in the models only marginally changed the estimates, suggesting that the effects could not be explained by reduced organochlorine exposure among women giving birth to live born children.

Another problem that potentially may affect the results is the low participation rate, especially in the Ukrainian population. However, we have no reason to believe that participation in Ukraine is related to the exposure, since the exposure level is unknown at the time of entering the study, and therefore the limited participation could at most limit the external validity of the study, if the participants differed from non-participant. The internal validity of the exposure-response associations would not be affected by such self-selection.

The proportion of previous pregnancies reported ending as a fetal loss was in the range of 18-25% in Ukraine and Greenland, which is in the range of previous studies on the prevalence of self-reported fetal loss [25]. However, the proportion of previous pregnancies ending as a fetal loss was higher in the Polish population compared to the other included populations, which is probably reflecting the recruitment situation in Warsaw. This population differs from the other populations by recruitment from maternity schools causing a higher participation of women with previous loss. However, ban of induced abortions in Poland contrary to the other countries may also increase the rate of self-reported spontaneous abortions, where induced abortions may be reported as spontaneous abortions. Therefore, especially the Polish data should be interpreted with caution. However, removing the Polish data from the overall comparisons only marginally changed the estimates in the combined analyses across countries.

The present study is part of a larger study on fertility in European and Inuit populations, and therefore the present study was not specifically designed to address the question on the potential effects of organochlorines on fetal loss. Our results were not able to conclude unequivocally whether organochlorines are involved in fetal loss. Therefore, studies with collection of serum samples prior to the pregnancy onset and follow-up on the outcome of the pregnancies similar to the study by Venners et al 2005 [15] on DDT and DDE are warranted for other organochlorine compounds or other compounds with suspected reproductive toxicity.

## Conclusions

In conclusion, the combined data across countries suggests that high levels of CB-153 and p,p'-DDE may be associated with increased risk of fetal loss and recurrent loss. This result needs cautious interpretation due to inconsistencies between countries and lack of clear exposure- response associations.

## List of abbreviations

BMI: Body mass index (kg/m^2^); CB-153: 2,2',4,4',5,5'-hexachlorbiphenyl; CI: 95% confidence interval; DDT: dichlorodiphenyltrichloroethane; OR: Odds ratio; POP: persistent organochlorine pollutants. p,p'-DDE: 1,1-dichloro-2,2-bis(p-chlorophenyl)ethylene; TCDD: 2,3,7,8- tetrachlorodibenzo-p-dioxin; TEQ: 2,3,7,8- tetrachlorodibenzo-p-dioxin equivalent.

## Competing interests

The authors declare that they have no competing interests.

## Authors' contributions

GT was involved in the study design, drafted the manuscript, and is leading the correspondence with coauthors and publishers. AMT was involved in study design, revision and final approval of the manuscript. BJ was involved in chemical analysis, revision and final approval of the manuscript. HSP was involved in data collection, revision and final approval of the manuscript.

JKL was involved in data collection, revision and final approval of the manuscript. VZ was involved in data collection, revision and final approval of the manuscript. JPB designed the original study and was involved in revision and final approval of the manuscript. All authors have read and approved the final manuscript.
